# A single-tube-braided stent for various airway structures

**DOI:** 10.3389/fbioe.2023.1152412

**Published:** 2023-03-16

**Authors:** Xin Tong, Yongkang Jiang, Fei Mo, Zhongqing Sun, Xiaojun Wu, Yingtian Li

**Affiliations:** ^1^ Shenzhen Institute of Advanced Technology, Chinese Academy of Sciences, Shenzhen, China; ^2^ School of Mechanical and Electrical Engineering, Xi’an University of Architecture and Technology, Xi’an, China; ^3^ School of Automation, Beijing University of Posts and Telecommunications, Beijing, China

**Keywords:** airway stent, photocurable stents, braiding method, stent customization, *ex vivo* experiments

## Abstract

**Background:** Airway stent has been widely used in airway procedures. However, the metallic and silicone tubular stents are not customized designed for individual patients and cannot adapt to complicated obstruction structures. Other customized stents could not adapt to complex airway structures with easy and standardized manufacturing methods.

**Object:** This study aimed to design a series of novel stents with different shapes which can adapt to various airway structures, such as the “Y” shape structure at the tracheal carina, and to propose a standardized fabrication method to manufacture these customized stents in the same way.

**Methods:** We proposed a design strategy for the stents with different shapes and introduced a braiding method to prototype six types of single-tube-braided stents. Theoretical model was established to investigate the radial stiffness of the stents and deformation upon compression. We also characterized their mechanical properties by conducting compression tests and water tank tests. Finally, a series of benchtop experiments and *ex vivo* experiments were conducted to evaluate the functions of the stents.

**Results:** The theoretical model predicted similar results to the experimental results, and the proposed stents could bear a compression force of 5.79N. The results of water tank tests showed the stent was still functioning even if suffering from continuous water pressure at body temperature for a period of 30 days. The phantoms and *ex-vivo* experiments demonstrated that the proposed stents adapt well to different airway structures.

**Conclusion:** Our study offers a new perspective on the design of customized, adaptive, and easy-to-fabricate stents for airway stents which could meet the requirements of various airway illnesses.

## 1 Introduction

Airway illnesses, such as tracheobronchomalacia (TBM) and tracheobronchial stenosis (TBS), may cause the patients to cough, wheeze, apnea and sometimes lead to profound airway obstruction which will threat patients’ life (Torre et al., 2012; Mitchell et al., 2014). TBM and TBS are sometimes congenital airway malformations, but more often caused by surgical trauma, tumor compression, anastomotic hyperplasia after lung transplantation, and extrinsic compression (Carden et al., 2005; Wright et al., 2019; Xiong et al., 2019). In clinical practice, the diseases are mainly treated by tracheostomy based long-term mechanical ventilation, aortopexy, suspension, and airway stenting (Torre et al., 2012; Mitchell et al., 2014; Fraga et al., 2016; Hysinger and Panitch, 2016). Due to the advantages in non-invasive properties during surgery and rapid post-surgery recovery, airway stenting procedures have been widely adopted to keep the airway open (Ha et al., 2019; Xiong et al., 2019).

To design airway stents used for the above-mentioned clinical practices, several requirements have to be met. First, the diameter and the radial stiffness of the airway stents must be systematically optimized, to keep the airway open and reduce the risk of unnecessary complications simultaneously. Oversized stent or too large radial stiffness will cause the airway tissue injured, e.g., mucosal ischemia, while undersized stent or too weak radial stiffness will result in migration inside airways (Murgu and Laxmanan, 2016). Second, the stent must own sufficient fatigue strength to avoid fracturing when suffered from the periodical contraction during breathing and sometimes coughing (Folch and Keyes, 2018). Finally, to avoid mucus plugging, the stent should minimize its impediment on the mucociliary clearance, so that cilia-mediated mucus can flow through the stented region (Bhora et al., 2016; Murgu and Laxmanan, 2016).

Over decades, airway stents has been constantly studied (Avasarala et al., 2019; Guibert et al., 2019; Guibert et al., 2020; Mathew et al., 2020; Paunović et al., 2021; Soriano et al., 2021; Ratwani et al., 2022). The most widely used airway stents in clinical procedures are self-expandable metallic stents (SEMS). They are designed into a series of standard dimensions and fabricated by laser cutting technique. The SEMS are standard-sized stents, but the sizes and structures of different patients vary a lot. Thus, this mismatch of the standard stents and non-standard patients’ airway make it difficult for patients to select proper stents during procedures. In addition, the sharp edges of SEMS will cause mucosal trauma of the airways, inducing the growth of granulation tissues which eventually requires invasive procedures to remove the stents (Dasgupta et al., 1998; Saad et al., 2003; Almadi et al., 2017; Folch and Keyes, 2018). Silicone rubber tubular stents, compared to the metallic stents, are much safer when deployed into patients’ airways due to their intrinsic soft properties. However, mucus plugs or even pneumonia may occur because the silicone stents will fully cover the airways and further block the secretions cleaning (Saji et al., 2010; Sökücü et al., 2020). To create a non-standard stent, there were trials to suture several silicone rubber tubular stents together to match complicated airway structures, such as the “Y” shape structure located at the tracheal carina. But the stents are too complicated during fabrication and still difficult to match the airway sizes perfectly (Long, 1988; Majid et al., 2012).

In the previous work, an *in vivo* molded airway stent was designed. The thermoplasticity-based single helical stent would not induce the growth of the granulation tissue and avoid blocking the mucociliary clearance, and its diameter can also be adjusted during clinical procedures (Mencattelli et al., 2021). The limitation of this work is that the single helical structure cannot adapt to complicated branches. For example, when the stent is required to support airway tissues at the tracheal carina, we had to implant three single helical stents individually instead of a Y-shape stent, which brings unnecessary complexity to clinical procedures (Dutau et al., 2004; Madan et al., 2016; Sehgal et al., 2017). Besides, the single helical structure has only one contact point at each cross-sectional area, limiting the support effect in the airway in some cases.

**FIGURE 1:**
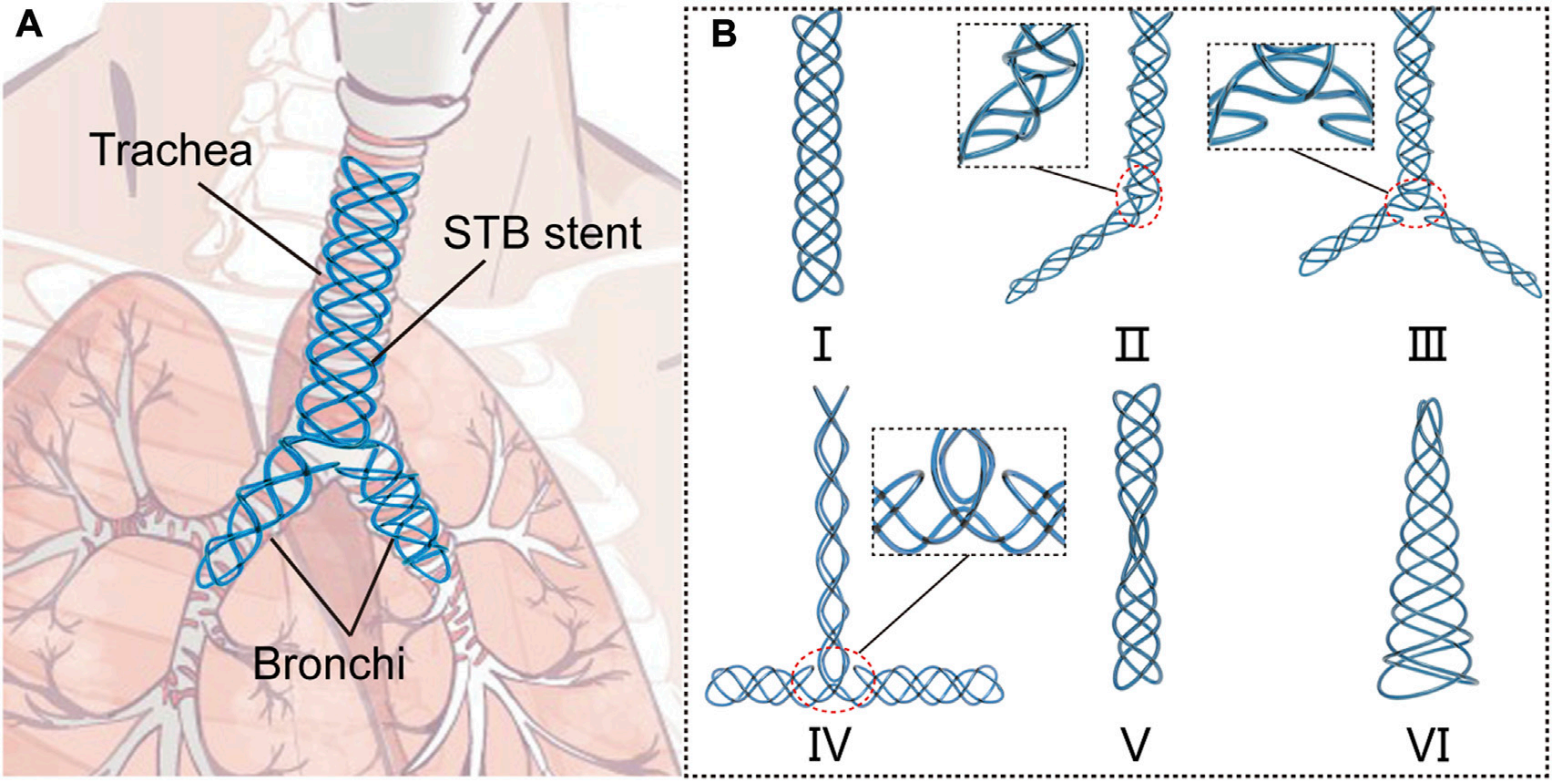
Concept of the single-tube-braided airway stent. **(A)** A schematic diagram, in which a Y-type single-tube-braided (STB) stent is implanted into the trachea and bronchi. **(B)** 6 types of STB stents for various airway structures: Type Ⅰ stent, a straight stent for central airway obstruction. Type Ⅱ stent, a L-shape stent to support trachea and one main bronchus. Type Ⅲ stent, a Y-shape stent to support trachea and both main bronchi. Type Ⅳ stent, a T-shape stent for tracheal intubation procedures. Type Ⅴ stent, an hourglass stent for tracheostenosis. Type Ⅵ stent, a conical stent for the disorder at the end of the airway.

In this paper, we proposed a novel series of stents which can be fabricated by the same braiding method using a single soft tube, and we named this type of stents as single-tube-braided stent (STB stent) (Figure 1A). The stents are photocurable, and their diameters can be customized during the clinical procedures. The STB stents are designed to exhibit different shapes in order to adapt to varied airway structures for various airway illnesses. Altogether six different shapes of the stents (Figure 1B) were demonstrated based on clinical requirements. Type Ⅰ to III stents are designed for malacia located at tracheal, bronchi and/or tracheal carina. Type IV stent can be used for tracheal intubation procedures, while Type V to VI stents could provide supports to different types of TBS. In addition to design and fabrication, we also established a theoretical model to illustrate the relationship of radial stiffness and radial deformation of the stents. Experiments were conducted to verify the model and evaluate the mechanical properties of the stents, including compressive tests and water tank tests. Then, the supporting behaviors were evaluated in phantom experiments. Finally, functional demonstrations were presented by stenting the proposed stents in the *ex vivo* trachea and bronchi harvested from a swine.

## 2 Material and methods

### 2.1 Stent design

#### 2.1.1 Design strategy

The design strategy of STB stents is described in this section. The STB stents are designed to be braided by a single tube filled with UV-curable polymer. The tube we selected is a biocompatible silicone tube, and the softness brought by the silicone material promise the stent to exhibit easy deformation and adaptation to the airway structures. To provide sufficient supporting force to the airway, the radial stiffness is the key design criteria. In this work, the UV polymer filled into the silicone tube is liquid before curing but will turn to rigid material after ultraviolet radiation. In view of the shortcoming of the *in vivo* molding stents, the STB stent is designed as multi-helix structure, to provide sufficient supporting force and contact points to the airways. From the perspective of design, five conditions must be fully met in this work.

First and foremost, the stent must be braided by one single tube, to provide customized stents for various airway illnesses, and the stent shall be composed of 2m helixes. Then, a left-hand helix and a right-hand helix with the same number of coils, which is symmetric about X-Y plane, are braided as a “curve group” structure defined in this work. The structures are illustrated by purple and blue curves in Supplementary Figure S1A. Third, to provide supporting forces to airways evenly, m pairs of “curve group” are designed to distribute uniformly in circumferential. Since single curve group structure cannot be evenly distributed and thus the number of m should be larger than 1. Fourth, a start point and an end point for each helix shall be defined, and the start point of later helix shall coincide with the end point of the former helix. At last, the start point of the first helix must coincide with the end point of the last helix, as shown in subfigure II of Supplementary Figure S1B. Because curve group structures have to be evenly distributed, the start point and end point of a single curve group structure have to locate at the two ends of the arc of the projected circle, whose length is 1/m+N or $\left(m-1\right)/m+N$ of the circumstance, where N is any natural number representing N full circles, as shown in Supplementary Figure S1C.

Although the following theory can be used to design the proposed stents with more than three pairs of curve group structures, the number of curve group structures m are selected to be 2 in this work. This is because the stents are required to avoid the impediment of mucociliary clearance, the contact area between stents and airways shall be minimized. Therefore, the stent in this work only consists of two curve group structures, indicating four helixes in total.

#### 2.1.2 Number of coils for the stent

For a single curve group structure, the two helixes must be symmetric, so the pitch and diameter of the helix are the same and the only parameter determining the length is number of coils for each helix n . Because they are connected to each other, the turning point shall locate at the middle points of the arcs formed by the start and end points. Therefore, the number of coils for each helix n could be written as a dataset when we have decided the value of curve group is m , the dataset and each data element in the set is expressed as Eq. 1:

$$N_{m}=\left\{n|n=\frac{N}{2}+\frac{k}{2m}\right\}$$ (1)

where k equals to 1 or m−1 .

**FIGURE 2:**
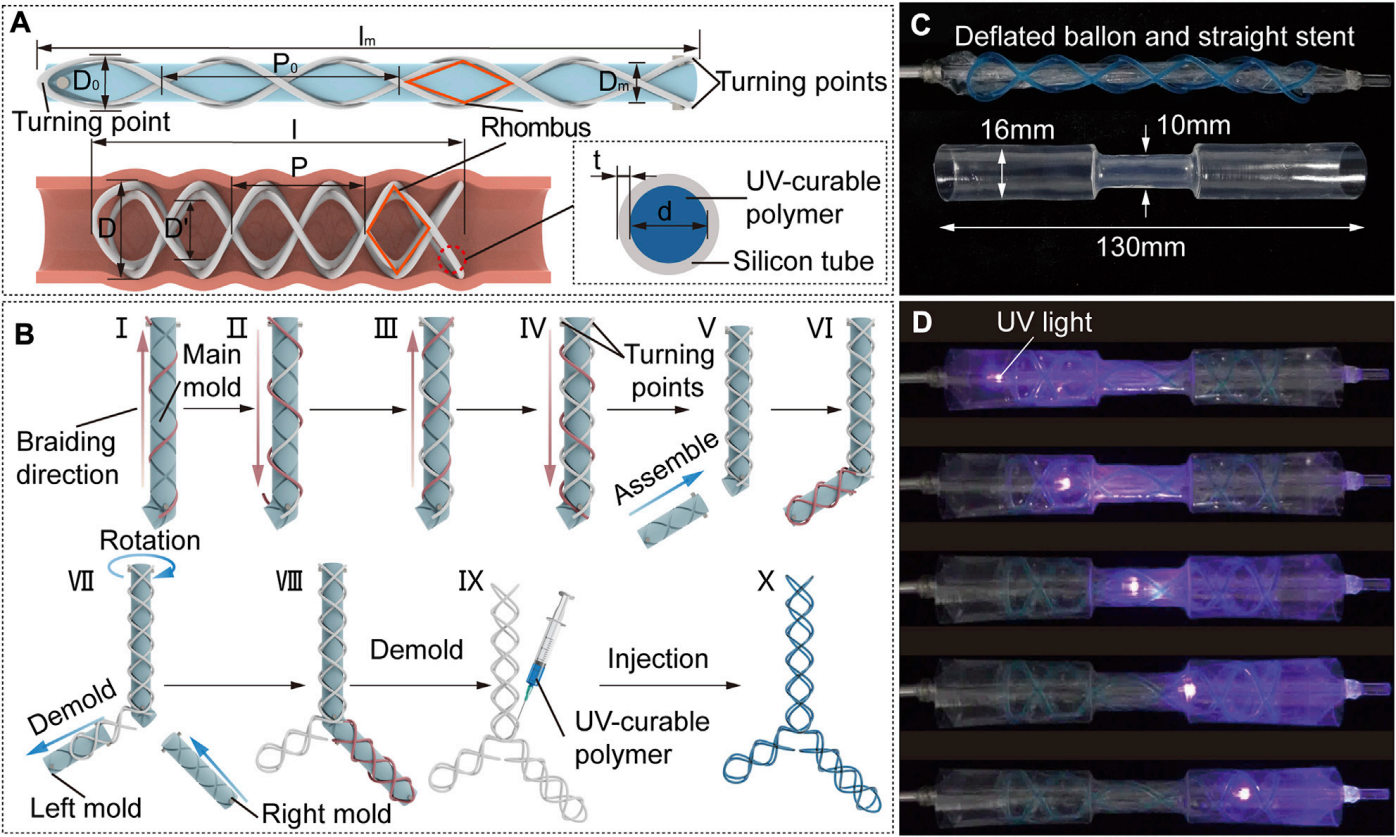
STB stent design strategy, fabrication and curing process. **(A)** Design parameters of an uncured STB stent on a mold (top), a cured STB stent in an airway (bottom left), and inner structure of the STB stent (bottom right). **(B)** Fabrication steps. A silicone rubber tube is manually winded along the grooves on the mold. Steps I to IV sequentially described the steps to wind the four helixes, and the tube highlighted in red represents the ongoing operation in these steps. Steps V to VIII separately illustrates braiding the second and the third branches of the stent. Step IX presents the injection of the UV-curable polymer. The ready-to use stent is shown in step X. **(C)** Initial straight stent on a balloon catheter, and a phantom tracheal model with a simulated stricture in the middle. **(D)** Curing process. The uncured stent is delivered into the model across the area of the stricture. Expand the stent radially by balloon dilation to conform with the model. Cure the stent with UV light provided by an optical fiber.

Before deciding the actual number of coils, the relationship between the number of helical coils and the parameters of airways shall be expressed.

$$n_{theory}=\frac{l}{P}$$ (2)

where $n_{theory}$ is the calculated helical coil number according to the length of diseased airway, P is the required pitch of helix after the stent is expanded in the airway, which will be discussed in Section 2.3.2, l is the length of the airway region need to be supported (Figure 2A).

However, $n_{theory}$ is the minimum value in theory and may not equals any one data element n in the dataset $N_{m}$ . Therefore, the number of coils for each helix in practice ( $n_{practical}$ ) should be no smaller than $n_{theory}$ to ensure a sufficient supporting, and the value should be selected from the dataset $N_{m}$ . Therefore, Eq. 3 expresses the way to select $n_{practical}$ .

$$\left.n_{practical}=\min(N_{m}(find(N_{m}\geq N_{theory})\right)$$ (3)

#### 2.1.3 Pitch and diameter of the stent

Once the number of helical coils of STB stents have been determined, the rest works to conduct is to decide the pitches and diameters of the stents. The four helixes of the stent are bonded to each other at their interaction points exhibiting numbers of rhombic structures (Figure 2A), which will keep the number of coils as a constant. The determination of the parameters starts from the molding process.

The stents will be winded onto the mold for consistency, so the diameter of the mold plus the twice the diameter of the tube shall be smaller than the diameter of human main bronchi (12 mm in general). The diameter shall be as small as possible, but not too small to unnecessarily enhance the difficulty in manufacturing.

Since we have the diameter of mold $D_{m}$ and the actual number of helical coils $n_{real}$ , we can easily obtain the pitch of mold $P_{0}$ by Eq. 4 (detail derivate process can be found in Supplementary Material “Derivation processes of Equation 4” and Supplementary Figure S2).

$$P_{0}=\sqrt[{2}]{\pi^{2}\left({D^{\prime }}^{2}-D_{m}^{2}\right)+P^{2}}$$ (4)

where $D^{\prime }=D-4\left(d+2t\right)$ is the inner diameter of expanded STB stent in airway, d and t are the inner diameter and thickness of the silicone tube used to fabricate the stents, and D is 3 mm larger than the trachea of human in diameter (15 mm–20 mm in general) to produce a similar preload against the trachea and prevent the stents from migration.

The length of mold $l_{m}$ can be calculated by $l_{m}=n_{practical}P_{0}$ , where $P_{0}$ is the pitch of the initial STB stent as shown in Figure 2B.

### 2.2 Stent fabrication and curing process

Here, the materials we chose, the fabrication method and curing process are described in this section. In this work, we selected a silicone tube with a thickness of 0.15 mm and the UV-curable polymer we selected with high elastic modulus (about 1,790 MPa measured with MARK-10, Mark-10 Corporation, NY, through a standard uniaxial extension test) and relatively low creep.

To braid the proposed STB stent of diverse shapes, we designed a modular molding technology (Figure 2B). The modular molds can be assembled into different configurations to fabricate different stents. The whole set of molds comprise main mold, left mold, and right mold with grooves on each of them, which is manufactured by a 3D printer (Ultimaker S3, Ultimaker B.V., MA). The grooves on the mold are braiding tracks to guide the braiding of the tube.

The braiding process is described in detail in Figure 2B. All the stents begin with winding the tube on the main mold, which are used to fabricate Type I, V and VI stents as shown in Figure 1B. The steps I to IV sequentially describe the steps to wind the four helixes, and the tube highlighted in red represents the ongoing operation in these steps. The whole braiding process is presented in Supplementary Video S1. The fabrication of other types of stents is built on the straight stent and requires the assembly of right and/or left mold onto the main mold. Step V illustrates assembling the left mold onto the main mold to form the L-shape mold, which is used to fabricate Type II stent. In step VI, the first four steps shall be repeated in sequence to braid the second branch of the stent. To fabricate the Type III and IV stents, the left mold should be first demolded and the right mold is then assembled onto the main mold as shown in step VII;. In step VIII, the second branch of the stent is braided by the above-mentioned steps. In step IX, the stent is demolded, and the UV-curable polymer is injected into the tube. The ready-to use stent filled with polymer is shown in step X.

The curing process is described in detail. The ready-to-use stent will be placed on a balloon catheter, with which the stent will be delivered to the airways. We used an hourglass-shape transparent pipe to represent the trachea with stricture, which was designed to be consistent with the anatomical shape of an adult trachea. The balloon catheter and pipe are shown in Figure 2C. As for curing process, the stent positioned on the uninflated balloon catheter is first delivered into the pipe. Then the balloon is inflated to expand the stent so that the stent could adapt to the varied pipe diameters. After that, we keep the inflation and insert an optical fiber connected to a UV-light source through the central lumen of the balloon catheter, to deliver UV-light to cure the polymer. The end of UV optical fiber can be positioned at different points along with the balloon to ensure fully curing of the stent. Finally, we deflate the balloon and remove the catheter, leaving the cured stent in the pipe (Supplementary Video S2).

### 2.3 Modelling and experimental method

In this section, we established a model to investigate the radial stiffness of the STB stents, and meanwhile we performed a series of experiments to investigate their mechanical properties. The deformation of the STB stent under uniformly distributed loading was studied in the first set of experiments, to verify the model. The second set of experiments investigated the fatigue performance of STB stents over time under radial loading (water pressure at a depth of 10 cm). The modeling and experiments are described below.

#### 2.3.1 Model

The stents used in clinical procedures are usually oversized than the airways to prevent the stent from migration and hence suffer from compressive forces due to the elasticity of the tissue. Breathing and coughing of patients can further improve the compressive force. We established a theoretical model to establish the relationship between the compressive force and the radial deformation of the STB stent. This model shows how the parameters influence the stiffness of STB stents, such as the cross-section area of cured polymer, diameter D and helical angle of STB stent.

**FIGURE 3:**
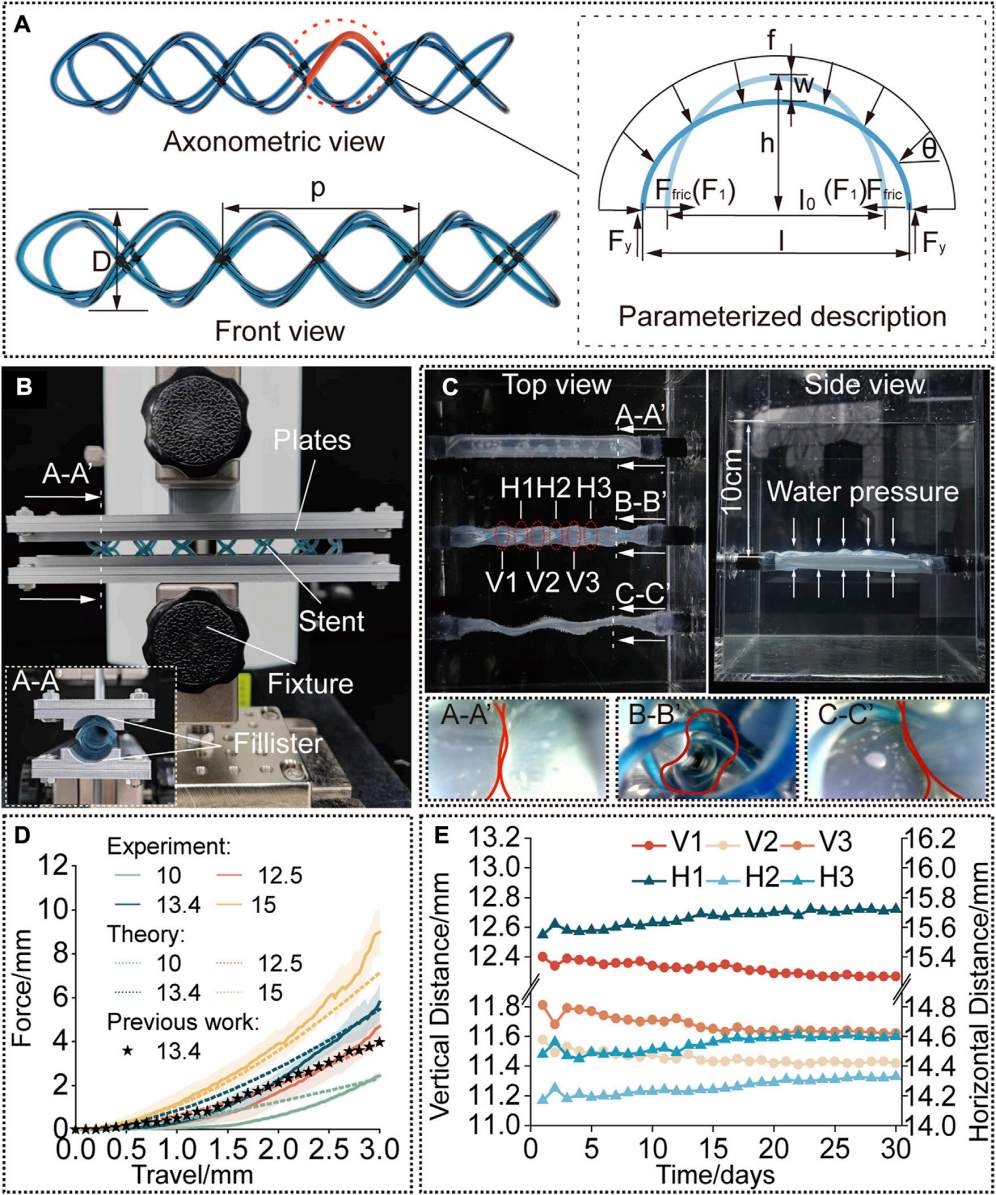
Modelling and characterization. **(A)** A rendered graph of a straight stent and a diagram of its deformation analysis. **(B)** Compression test setup. The cured stent was positioned between the two plates with fillisters and compressed by a force-measuring platform. **(C)** Water tank test setup. The stent is kept inside the phantom tracheal model at 37°C and a water pressure at a depth of 10 cm was applied. **(D)** Compression force *versus* travel distance. Experimental results compared to the predicted results to verify the model and compared to the results for the single helical stent with the same parameters. **(E)** Diameters of the stent [interpreted by the vertical and horizontal distances as shown in **(C)**] *versus* time (30 days).

Based on the design configuration, we assume that the force applied on all helixes are the same. To simplify the calculation, half coil of a single helix is chosen for analysis (Figure 3A). The curve can be considered as half of an elliptical beam, which will be pressed by a uniformly distributed loading force f . Then, we can express the compressed ellipse by Eq. 5:

$$\frac{4x^{2}}{l^{2}}+\frac{y^{2}}{{\left(h-w\right)}^{2}}=1$$ (5)

where $h=\frac{D}{2}$ is the equation of minor axis of the compressed ellipse, w is the deflection of middle point on the beam, and the major axis of the compressed ellipse $l=\sqrt[{2}]{D^{2}+{\left(\frac{P}{2}\right)}^{2}}$ .

The perimeter of the semi-ellipse is kept constant due to the inextensibility of the cured polymer, we can get the relationship of the axes before and after compression:

$${\left(\frac{l}{2}\right)}^{2}+{\left(h-w\right)}^{2}={\left(\frac{l_{0}}{2}\right)}^{2}+h^{2}$$ (6)

where, $l_{0}$ is the initial major axis of ellipse. This equation is calculated based on the approximated calculation formula of the perimeter of ellipse, which is generally written as $L=\pi \sqrt[{2}]{2\left({\left(l_{0}/2\right)}^{2}+h^{2}\right)}$ . In this equation, L is the perimeter of ellipse (Muir, 1902).

The horizontal and vertical component of the uniformly distributed loading force f is expressed as Eq. 7

$$f_{x}=fcos\theta ;f_{y}=fsin\theta$$ (7)

where,

$$\begin{array}{c} \left\{\begin{array}{c} \sin\theta =-\frac{\left(h-w\right)x}{\sqrt[{2}]{{\left(\frac{l}{2}\right)}^{4}-x^{2}\left[{\left(\frac{l}{2}\right)}^{2}-{\left(h-w\right)}^{2}\right]}}\\ \cos\theta =\frac{\frac{l}{2}\sqrt[{2}]{{\left(\frac{l}{2}\right)}^{2}-x^{2}}}{\sqrt[{2}]{{\left(\frac{l}{2}\right)}^{4}-x^{2}\left[{\left(\frac{l}{2}\right)}^{2}-{\left(h-w\right)}^{2}\right]}} \end{array}\right. \end{array}$$

Then, we can obtain the reaction force by Eq. 8:

$$F_{y}=\int_{-\frac{l}{2}}^{0}f_{y}dx$$ (8)

The friction between the beam and the internal wall of the airway was assumed as below (the detailed explanations refer to Supplementary Material “Explanation for Equation 9” and Supplementary Figure S3):

$$F_{fric}=\frac{2\mu AEw^{2}}{L*l}$$ (9)

where μ is the friction coefficient, A is the cross-sectional area of the beam.

Then, the moment applied to point $x_{0}$ , which could be at any point on the beam, can be written as:

$$M=F_{y}\left(x_{0}+\frac{l}{2}\right)-\int_{-\frac{l}{2}}^{x_{0}}f_{y}\left(x_{0}-x\right)dx-\int_{-\frac{l}{2}}^{x_{0}}f_{x}\left(y\left(x_{0}\right)-y\left(x\right)\right)dx-F_{fric}y\left(x_{0}\right)$$ (10)

In general, the equation of deflection w can be expressed as Eq. 11.

$$EIw^{\prime \prime }=-M$$ (11)

Therefore, we can obtain the relationship between deflection w and the uniformly distributed loading f by substituting Eq. 10 into Eq. 11 and integrating the corresponding expression.

#### 2.3.2 Experimental method

The stiffness of the airway stent shall be strong to provide sufficient radial support to keep the airway open during breathing and sometimes coughing. Therefore, we conducted a few sets of experiments. The experiment aims to prove that the stents fabricated with the selected tube own sufficient stiffness for clinical procedures.

### 2.4 Radial stiffness

First, the stents were designed with the diameter of 13.4 mm and pitch of 18.6 mm, to compare the radial stiffness to our previously published work (Mencattelli et al., 2021) which had already proved its practicability in *in vivo* animal experiments. Second, the cured diameter of the stents was chosen as the variate, whose initial diameter before balloon dilation was kept the same, to investigate the influence of diameter on the radial stiffness in response to compressive loading, and the results were also compared to the results predicted by the proposed model. The parameters of the STB stent before curing were calculated according to Eq. 4. To conduct the tests, the stents were radially compressed by a displacement of 0 mm–3 mm, which is referred to the research work (Mencattelli et al., 2021). Force data was collected 20 points per second, and each trial were repeated for three times. The results are presented in Figure 3D.

The testing platform consists of a lifting platform with a force gauge (MARK-10, Mark-10 Corporation, NY) and two parallel plates with curved fillisters, between which the stents are positioned, as shown in Figure 3B. The curvature of the fillisters matched the unloaded diameters of the stents (10 mm, 12.5 mm, 13.4 mm, and 15 mm) and each plate covered a circumferential angle of 20°C on each side of the stent. We assume that the deformation of the stents in the experiment is similar to that under radial loading. To simulate the slippery in airways, the stents and fillisters were lubricated (Multi-Use Performance Lubricant with Teflon, DuPont, NY).

### 2.5 Fatigue property

After implanted, the stents are suffering from continuously radial loads at body temperature over a long period of time. Therefore, to evaluate the performance of stents in a simulated environment, we performed water-tank tests. The proposed stents were delivered into a phantom tracheal models, which was placed under 37°C water (Figure 3C). According to reference, the continuous positive airway pressure (CPAP) to treat airway disorders has been proven effective, and the value of the applied pressure which is sufficient to maintain respiration equals to a water pressure at a depth of 5 cm–10 cm (Pizer et al., 1986; WEIGLE, 1990; Panitch et al., 1994).

The phantom tracheal model was casted with silicone with an inner diameter of 10 mm and a thickness of 0.2 mm (The detailed material constants based on Yeoh model are: $C_{1}=1.0\times 10^{-1},C_{2}=1.69\times 10^{-1},C_{3}=2.66\times 10^{-4}$ ). According to (Ha et al., 2019), the properties of the silicon had been proved with biaxial testing experiments to exhibit similar mechanical property to the actual trachea. This tracheal model was easier to collapse radially because of its soft property. This means we simulated a worse case of TBM, when the tracheal cartilages completely lose their functions and cannot provide supports to the tracheal tissue, to evaluate the performance of our stents (see Supplementary Figure S4).

As a comparison, three phantom tracheal models were side-by-side positioned in a water tank under a depth of 10 cm. The ends of the phantoms were glued and sealed to rigid tubes, which passed through the holes in the wall of the tank. To produce a similar preload against the trachea, the diameter of stents implanted in the tracheal model is 3 mm larger than the diameter of the phantom. As shown in Figure 3C, an STB stent was cured inside the middle phantom with a cured outer diameter of 13 mm, a single helical stent whose diameter and pitch are the same with the STB stent was delivered into the bottom phantom, while the phantom on the top has nothing inside. The endoscopic views at the bottom of Figure 3C show that only the phantom with the STB stent did not collapse (Supplementary Video S3, Figure S5).

## 3 Results

### 3.1 Mechanical properties

The results show the radial stiffness of the stents were presented in Figure 3D, and the stiffness was interpreted by the external compressive force. It is obvious that a cured STB stent with a larger diameter exhibits a stronger ability to resist a compressive force. If compressed no more than 1.8 mm, the resistance ability for external compressive force of the STB stent, whose pitch is 13.4 mm and silicone tube is 1 mm in diameter, is similar to that of our previous single helical stent. However, when compressed displacement exceeds 1.8 mm, the resistance ability of STB stent is larger than that of the single helical stent. This means that the proposed stent has proved its resistance ability for external compressive force is large enough to meet clinical requirements (Ha et al., 2019; Mencattelli et al., 2021).

The results predicted with the model showed similar trend to the experimental results in Figure 3D which verified the effectiveness of the above-mentioned model. In the first stage of compression, the predicted results were a little bigger than the experimental results. The differences decreased with the increase of compressive displacement. In the second stage, the experimental results increased with an even faster rate and showed larger values than the predicted ones. The reasons behind are discussed here. 1) In the model, we assumed that the elliptical beam suffered a uniformly distributed load (Supplementary Figure S6A). However, due to the fabrication error in practice, the stents cannot always match well with the fillisters of plates (Supplementary Figures S6B). The mismatch made the uniformly distributed load a concentrated force and provided less constraints to the boundary of the stents. Therefore, in the first stage, the stents were easier to deform with a smaller compressive force. 2) After the stents were compressed for a few millimeters, the compressed stents fully contacted with the fillisters. If continue the compression, in theory, the compressed stents should elongate along the axial direction of the stents (Supplementary Figures 6C, 6E). But as illustrated in Supplementary Figure S6D, the stents would deform into the gap between the plates, resulting in irregular deformation. A large portion of the compression force would be transmitted to the region to induce the irregular deformation rather than compressing the stent as a whole. This may be the reason leading to larger forces than the predicted ones in the second stage.

Figure 3E shows the radial deformation of the STB stent over a period of 30 days. In each measurement, 6 sets of data were recorded, and they are three pairs of horizontal distances (H1, H2, and H3) and three pairs of vertical distances (V1, V2, and V3). The details are explained in Figure 3C; Supplementary Figure S7. The outer diameter of the STB stent decreased from 13 mm to 11.4 mm during the 30 days. The change of diameter is 1.6 mm, which is smaller than 3 mm stated above, and the stent could still maintain the airway open. Therefore, it is safe to conclude that the STB stent would not fail for 30 days even if it is stented in a worse simulated diseased airway.

### 3.2 Benchtop and ex-vivo experiments

**FIGURE 4:**
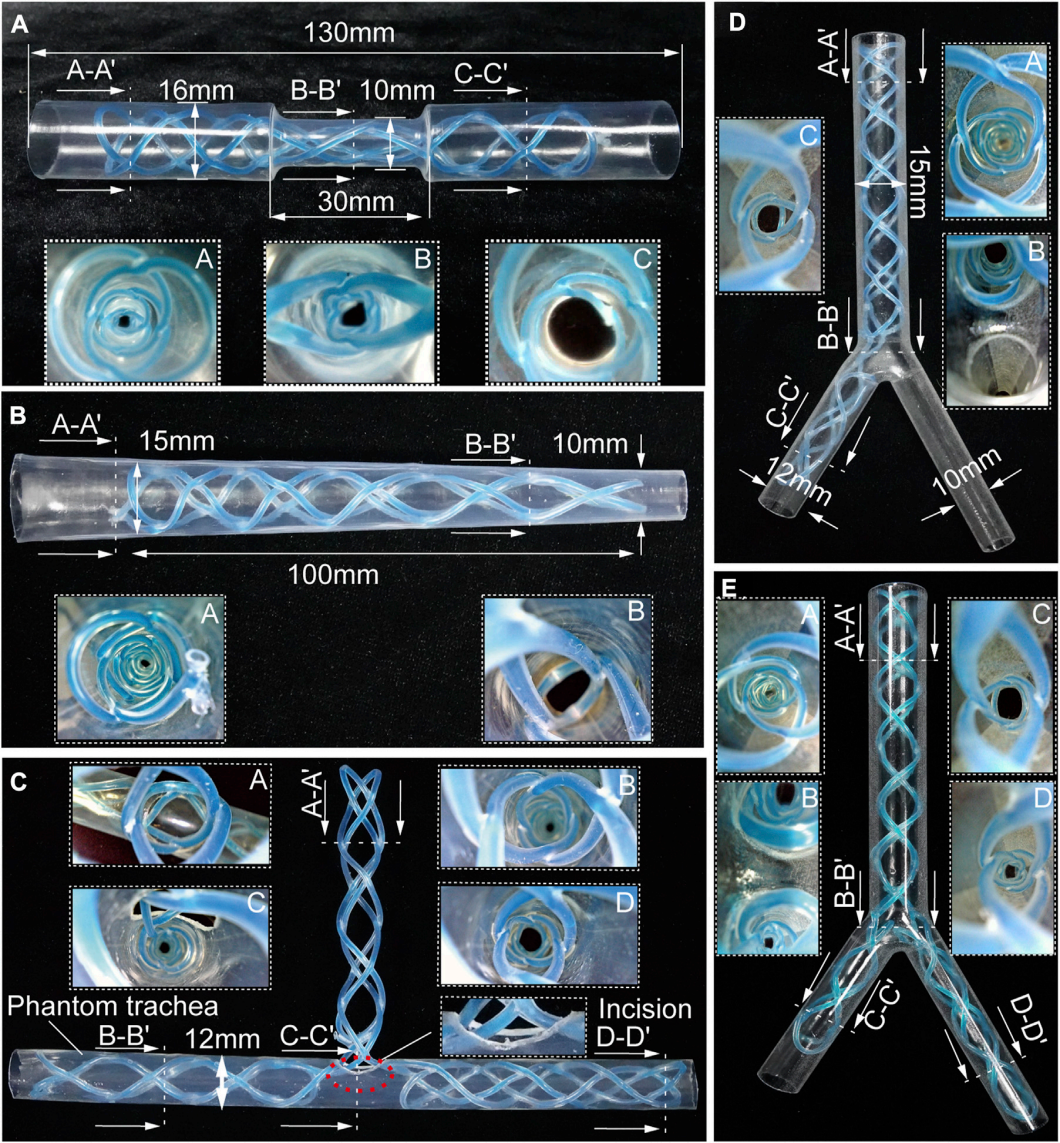
Benchtop experiments. Different shapes of the stents cured inside the corresponding phantom tracheal models with endoscopic views. **(A, B)** Molded straight stents inside tracheal phantom model with insets showing endoscopic views. **(C–E)** Molded complicated-shaped stents inside a phantom model of tracheal and bronchi with insets showing endoscopic views.

The STB stent proposed in this paper can be designed to produce different types of stents for various airway structures. To demonstrate stenting the STB stents in different airway structures, we designed, fabricated and cured the rest five types of stents, including stents in hourglass shape, conical shape, T shape, L shape, and Y shape. We also fabricated five different phantom airway models accordingly (Figure 4).

#### 3.2.1 Benchtop experiments

Airway stenosis shows diameter decrease in airways. Stenting in such an area is the variation of airway stents in diameter. The hourglass shape stent, illustrated in Figure 4A, was obtained by curing an initial soft straight stent in a tracheal model with a narrowed region to simulate stenosis. The tracheal model, which was fabricated by a heat shrinkable tube with a wall thickness of 0.5 mm, is 130 mm in length, with a outer diameter of 16 mm, while the narrowed region is 30 mm in length and 10 mm in outer diameter. Compared the diameters at point A and point C, the diameter at point B was much smaller, which is observed with an endoscope. This proved the stents adapted well to both the normal trachea model and the narrowed tracheal model.

Similarly, when the stenosis occurs at one end of airways, the conical stent will be a good choice. As shown in Figure 4B, a conical STB stent was cured in a conical tracheal model, and the endoscopic view clearly showed that the stent formed could match the inner wall of the phantom well. Therefore, it is safe to conclude that the straight STB stent could adapt to varied airway diameters.

The other three types of stents (T shape, L shape, and Y shape) investigated whether the proposed STB stent could provide support to different airway regions in more complicated scenarios. The T shape stent shown in Figure 4C was implanted into a tracheal model with incisions to simulate the functions of tracheostomy tubes. In Figures 4D, E, we 3D printed a transparent tracheobronchial model (transparent resin, Projet, SD system) to illustrate STB stent adapted to the trachea and bronchi at the same time. This 3D printed model with a thickness of 1 mm simulated a trachea with the outer diameter of 15 mm and the bronchi with outer diameters of 10 mm and 12 mm separately. The L shape stent and Y shape stent were delivered into and cured inside the model (Figures 4D, E). The results illustrates the proposed stents can be used for various complicated airway illness (Supplementary Video S4).

#### 3.2.2 *Ex vivo* evaluation

**FIGURE 5:**
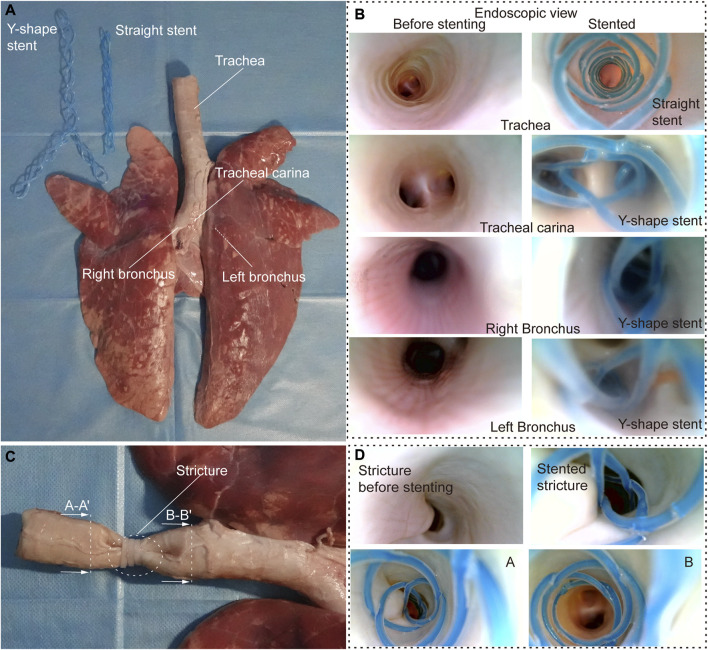
*Ex vivo* experiments. **(A)** *Ex vivo* swine airways and two types of stents used in the experiment. **(B)** Endoscopic view before (left) and after (right) stenting of the inner walls of the trachea, trachea carina, right and left bronchus. **(C)** Stricture created to simulate stenosis in the *ex vivo* trachea. **(D)** Endoscopic view before and after stenting in *ex vivo* trachea with stenosis.

To further evaluate the stenting performance of STB stents in real airways, we performed *ex vivo* experiments with trachea and bronchi harvested from a swine (Figure 5A). The inner diameters of the trachea and bronchi are 17 mm, 13 mm, and 12 mm respectively, and endoscopic inspection were performed as shown in Figure 5B.

We tested the stents in normal airways to validate their basic functions and the delivering procedures. We cured a straight and a Y-shape STB stent separately, and endoscopic inspection were performed. It is clear that the stents adapted to the inner walls of the trachea well, and no visual mechanical injuries caused by the deployment procedure was found (Figure 5B). After that, to simulate the trachea stenosis, we manually created a 2-cm length stricture in the middle of the *ex vivo* swine trachea, by externally compressing the trachea wall with three zip ties (Figure 5C). The stricture constrained airway and left only approximately 1/5 of its original inner cross-sectional area, which simulated a severe tracheal stenosis (upper left panel in Figure 5D). To simulate the clinical procedure, we delivered a straight STB stent covered the stricture area. The stent was delivered with the above-mentioned balloon catheter, which is also used to conduct balloon dilation to open the stricture area by a little bit. The dilated stricture reopened to approximately 2/5 of its original cross-sectional area. The stent was successfully cured in the area and could support the trachea to avoid future stenosis. The endoscopic inspection is shown in (Figure 5D) and (Supplementary Video S5).

## 4 Discussion

This paper proposed a single-tube-braided airway stents for various airway illness. The contribution of this work is summarized here. We explained the design strategy and fabrication method for the proposed stents and six types of stents have been prototyped to demonstrate their adaptation to different airway structures. We also established a model to predict the influence of the design parameters on the radial stiffness of the stents under external compression. Then, experimentally verifications were conducted to investigate the deformation of the stents in response to external loads. It showed that the cured STB stents could bear a compression force of 5.79N, which is 145% of what could be tolerated by the previous stents, and proved the proposed stent has met the clinical requirements on stent stiffness. In addition, the fatigue property of the proposed stents was tested, and the stent could keep the phantom tracheal model open when suffered continuously loads of 10-cm water pressure at body temperature for a period of 30 days. Moreover, benchtop experiments and *ex vivo* evaluations on swine trachea and bronchi were conducted to further validate the practicability and adaptation of the proposed stents.

Our stent, compared to the commercial self-expandable metallic stents (SEMS), would not cause injuries to the tissue, and the geometrical design would not induce the growth of granulation tissues which may require invasive procedures to remove the stents out of the airway. In terms of the comparison with silicon stents in clinical trials, our stents can reduce the impediment on mucus flow and has less possibility to cause mucus plugs. In addition, the proposed stent shares the same advantage with the previous work that the stents can be easily screwed out of airways to reduce the chances to hurt the tissues, but this stent could provide more reliable and uniform supports to the trachea than the previous work. Moreover, the proposed braiding method provides possibility to manufacture all types of airways stents with the same fabrication technique.

## 5 Limitations of the study

Our study demonstrated the potential of STB stents, but there are still limitations which require further investigation in future works. 1) Fabrication: To fabricate a STB stent, we manually braided the tube on a 3D printed mold, which is not a standard fabrication technique. The errors in fabrication will add inaccuracy to the theoretical prediction. Thus, an automatic braiding equipment shall be built to improve the fabrication precision in the future. 2) Modeling: In this paper, to simplify the modeling process, we chose half elliptical beam as the representative for analysis. We also made a few assumptions or used approximated calculations formulas. Even though the modeling showed similar results to the experiments, the accuracy of theoretical model is limited. 3) Experiment: It is normal to find both continual and fluctuant breathes in real breathing. But in the water tank tests, the silicone tube was placed under water with the inner space connected to the atmosphere. The experiment only provided static tests but no dynamic physiological conditions were simulated. 4) Animal test: Only *ex vivo* experiment was conducted in this work. To further evaluate the practicability of STB stents in clinic procedures, we still need to conduct *in vivo* experiments to test the proposed stents in future.

## Data Availability

The raw data supporting the conclusion of this article will be made available by the authors, without undue reservation.
