# Supplementary material for: A single-tube-braided stent for various airway structures
Source: Front Bioeng Biotechnol. 2023 Mar 16;11:1152412. doi: 10.3389/fbioe.2023.1152412 (PMC10060983; doi:10.3389/fbioe.2023.1152412)
Supplement: Supplementary file 2 [file Presentation1.pdf]

Supplementary Material

A single-tube-braided stent for various airway structures

Xin Tong,[1,2,†] Yongkang Jiang,[3,†] Fei Mo,[1] Zhongqing Sun,[1] Xiaojun Wu,[2,*] and Yingtian Li[1,*]

[1]Shenzhen Institute of Advanced Technology, Chinese Academy of Sciences, Shenzhen, 518055, China

[2]School of Mechanical and Electrical Engineering, Xi’an University of Architecture and Technology, Xi’an, 710055, China

[3]College of Mechanical and Electrical Engineering, Nanjing University of Aeronautics and Astronautics, Nanjing, 210016, China

*** Correspondence:** Xiaojun Wu, Yingtian Li
wuxiaojun@xauat.edu.cn, yt.li@siat.ac.cn

†These authors contributed equally to this work and share first authorship.

1 Supporting Information for Section 2.1 and 2.3

1.1 Derivation of Equation 4

The standard equation to calculate the length of a helix l is normally expressed as: $l = n\sqrt{(\pi d)^2 + p^2}$, where n is the number of coils for the helix, d is the diameter of the helix and p is the pitch of the helix as shown in supplementary figure 2.

Based on the standard equation, the equation to calculate the length of coils for the helix in this work is expressed by Equation 4 of the response, which is the same as the Equation 1_Supplementary:

$$l_{expanded} = n_{practical}\sqrt[2]{(\pi D')^2 + P^2} \qquad \text{(Equation 1)}$$

where, $l_{expanded}$ is the length of the expanded stent, $n_{practical}$ is the number of each helical coil, $D' = D - 4(d + 2t)$ is the diameter of expanded stent, and P is the pitch of expanded stent (figure 2A of the manuscript). Similarly, we can obtain the helix coil length on the mold:

$$l_{mold} = n_{practical}\sqrt[2]{(\pi D_m)^2 + P_0^2} \qquad \text{(Equation 2)}$$

Due to the length of each helix is a constant, we can find that $l_{expanded} = l_{mold}$, then we can get:

$$P_0 = \sqrt[2]{\pi^2(D'^2 - D_m^2) + P^2} \qquad \text{(Equation 3)}$$

1.2 Explanation for Equation 9

The friction is produced by the relative motion between the helix and the inner wall of the compressive mold. During the compression, the minor axis of the semi-ellipse would decrease which induced the deflection of middle point on the beam (w), and the major axis would increase from l_0 to l.

Due to the boundary constraints of the inner wall of the compressive mold, the stent rotates around the minor axis, which is constrained by the friction. Thus, it is clear that the direction of friction (F_{total}) between the helix and the inner wall of the mold is along with the axis of the stent (Supplementary figure 3). We decomposed the force into two components. The direction of the first component (F_1) is from one endpoint to the other endpoint of the beam of the ellipse. The direction of the second friction component (F_2) is vertical to the elliptical plane (X-Y plane), leading to the rotation of the stent instead of causing the deformation of the ellipse beam. For this reason, we just analyzed the first friction force named as F_{fric}.

The friction is affected by the normal force applied to the helix. But the normal force applied to the stent keeps changing either in the experiment or in clinical practice, due to the dynamic physiological activity and the rotation of the stent. This change of normal force is too complicated to predict accurately. However, we noticed that the deformation of the elliptical beam will apply a reaction force to the tissue, which will also induce a friction with the same value but opposite to the friction applied to the stent. Therefore, we took advantage of the deformation of the elliptical beam to estimate the equation of friction. It is clear that the cross-sectional area of the beam A, the change in deflection w, and the elastic modulus of the material E have positive correlation with the reaction force, while the perimeter of half elliptical beam $L/2$ is negatively correlated to the reaction force. In addition, we assumed that the value of $cos\alpha$ equals w/l. Due to the complexity of the stent structure and the difficulty in accurately calculating the value of friction during its deformation in practice, we estimated the friction F_{fric} is $\frac{2\mu AEw^2}{L*l}$. The assumptions and simplification make the model inaccurate, which is discussed in the limitation of this work.

2 Supplementary Figures

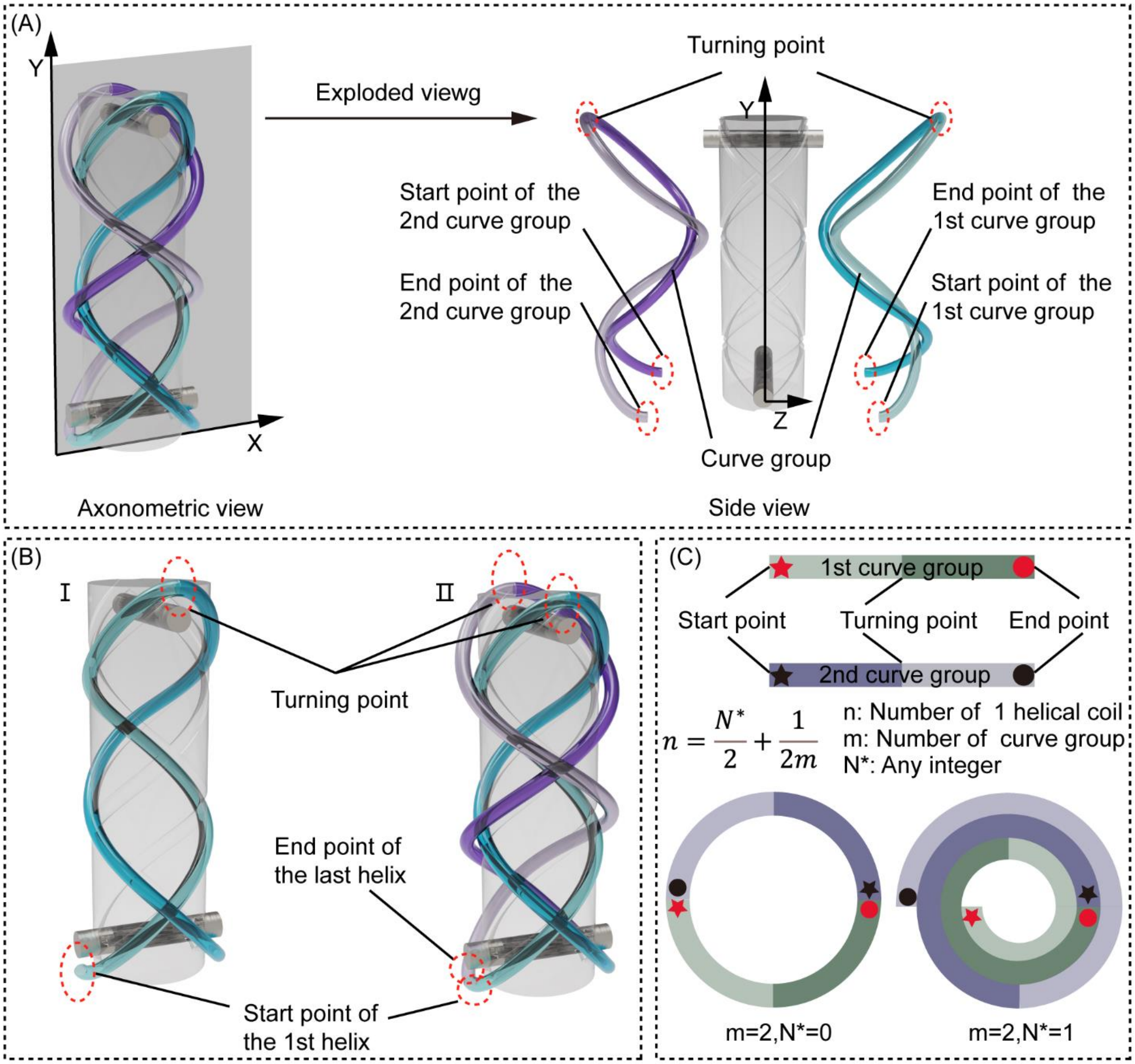

Supplementary Figure 1. Stents design and analysis. (A) Axonometric view and exploded view of the STB stent braided on the mold. (B) Illustration of the winding method and definition of the start point, end point and turning point of the curve group structures. (C) Schematic of projecting 2 pairs of curve group structures, and the conditions to meet based on the design strategy.

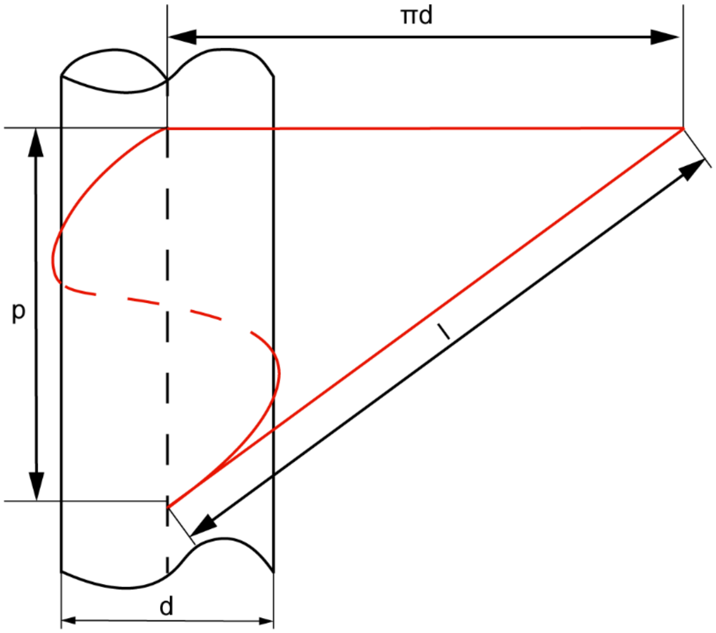

Supplementary Figure 2 Schematic of calculating the length of a standard helix

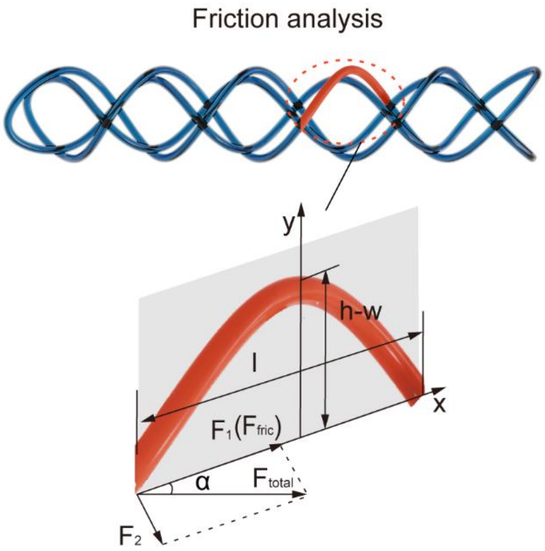

Supplementary Figure 3 Parameterized description of the friction on the chosen elliptical beam

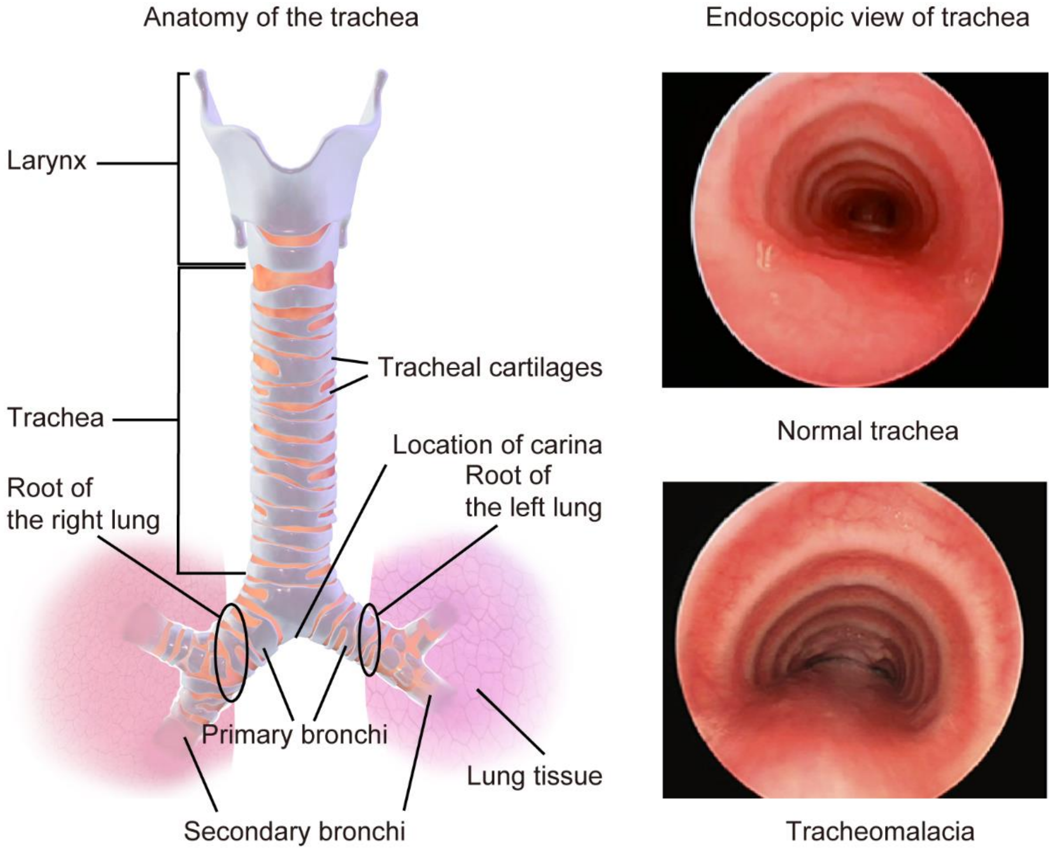

Supplementary Figure 4. Schematic of trachea and bronchi[1] and endoscopic views of normal trachea and sick trachea[2].

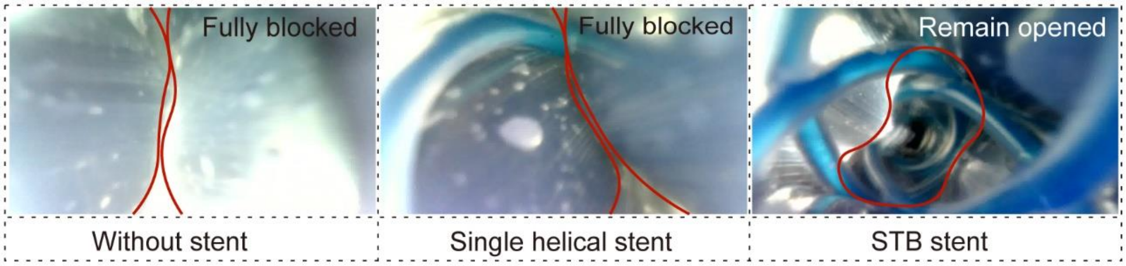

Supplementary Figure 5. Endoscopic view of stenting different stents in the phantom tracheal models which are placed inside water tank.

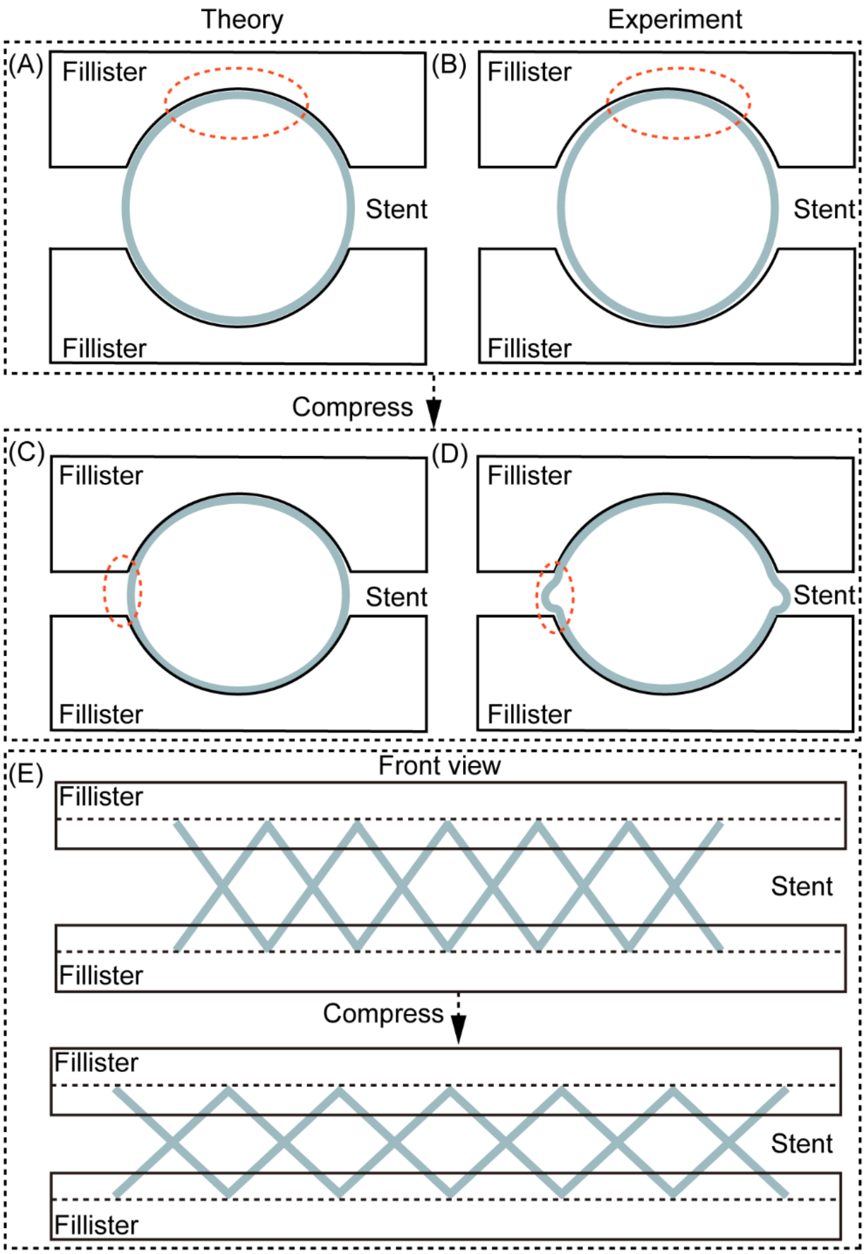

Supplementary Figure 6. Schematic of stent deformation under compression.

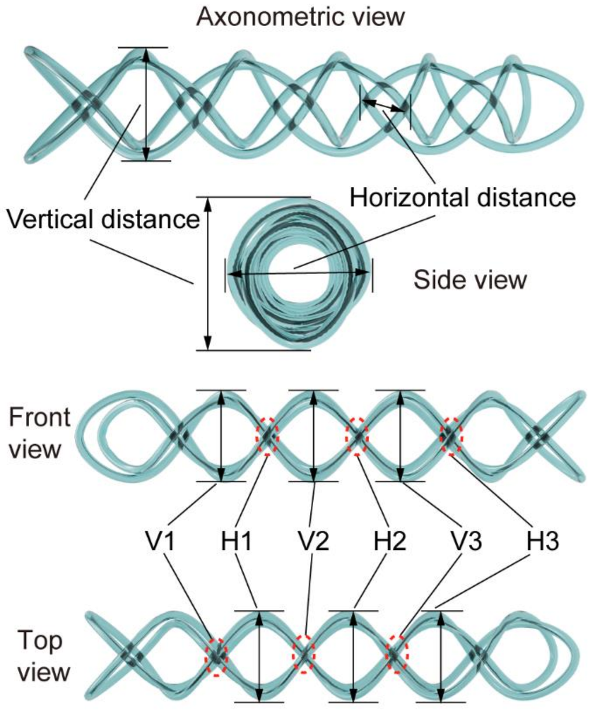

Supplementary Figure 7. Illustration of vertical distances and horizontal distances.

3 References

1. Blausen.com staff (2014). "Medical gallery of Blausen Medical 2014". WikiJournal of Medicine 1 (2).
2. Kamran A, Jennings R W. Tracheomalacia and tracheobronchomalacia in pediatrics: an overview of evaluation, medical management, and surgical treatment[J]. Frontiers in pediatrics, 2019, 7: 512.
